# Feeding Behavior of Finishing Pigs under Diurnal Cyclic Heat Stress

**DOI:** 10.3390/ani13050908

**Published:** 2023-03-02

**Authors:** Marllon José Karpeggiane de Oliveira, Marcio Valk, Antônio Diego Brandão Melo, Danilo Alves Marçal, Cleslei Alisson Silva, Graziela Alves da Cunha Valini, Pedro Righetti Arnaut, Joseane Penteado Rosa Gonçalves, Ines Andretta, Luciano Hauschild

**Affiliations:** 1 Department of Animal Science, School of Agricultural and Veterinarian Sciences, São Paulo State University (Unesp), Jaboticabal 14884-900, São Paulo, Brazil; 2 Department of Statistics, Universidade Federal do Rio Grande do Sul, Porto Alegre 91540-000, Rio Grande do Sul, Brazil; 3 Department of Animal Science, Universidade Federal do Rio Grande do Sul, Porto Alegre 91540-000, Rio Grande do Sul, Brazil

**Keywords:** circadian rhythm, feed pattern, light program, meal pattern, precision feeding, swine

## Abstract

**Simple Summary:**

Pigs reared in tropical climate areas are frequently exposed to high ambient temperatures. The increase in ambient temperature above thermoneutrality evokes behavioral changes that alter the feeding pattern of pigs, triggering a reduction in performance. In addition, the light program may also modulate the feeding behavior of pigs. Thus, data collected using electronic feeders were used to generate information on pig feeding behavior to identify anomalies that occurred due to variations in ambient temperature (cyclic heat stress) and after turning the lights on and off (light events). Our results indicated that cyclic heat stress disrupts the feeding circadian rhythm in finishing pigs. Pigs prioritized the feed intake in the coolest hours of the day. However, nocturnal cooling did not allow the pigs to fully compensate for the feed intake depression caused by heat stress. Furthermore, the lighting program affected the feeding pattern, increasing or decreasing the meal size when the lights were switched on or off, respectively. Understanding pig feeding behavior during cyclic heat stress and light events could improve feeding strategies, productivity, and animal well-being.

**Abstract:**

The impact of cyclic heat stress (CHS) and turning the lights on and off on pig feeding behavior (FB) was investigated. The FB of 90 gilts was recorded in real-time under two ambient temperatures (AT): thermoneutrality (TN, 22 °C) or CHS (22/35 °C). The day was divided into four periods: PI _(06–08 h)_; PII _(08–18 h)_; PIII _(18–20 h)_; and PIV _(20–06 h)_. Automatic and Intelligent Precision Feeders recorded each feed event for each pig. An estimated meal criterion (49 min) was used to calculate the FB variables. Feed behavior in both ATs followed a circadian pattern. The CHS reduced the feed intake by 6.9%. The pigs prioritized feed intake during the coolest hours of the day; however, nocturnal cooling did not allow the pigs to compensate for the reduced meal size due to CHS. The highest meal size and most of the meals were observed during the lighting-on period. The pigs reduced their interval between meals during PII and PIII. The lighting program increased the meal size when the lights were switched on and reduced the meal size when the lights were switched off. Thus, the dynamics of the FB were largely influenced by AT, whereas the meal size was affected by the lighting program.

## 1. Introduction

The improper thermal environment of housing facilities and lighting programs are detrimental factors that affect pig growth performance. Ambient temperature (AT) is related to the regulation of voluntary feed intake (VFI), triggering changes in feed behavior (FB). Finishing pigs exposed to high AT in cyclic heat stress (CHS) conditions reduce their VFI by 7.3% [1] and up to 50% in constant heat stress (HS) of 35 °C [2].

Reduction of VFI is a pig’s adaptive response in an attempt to decrease the metabolic heat production inherent to digestive and metabolic processes [3]. In addition to the impact on VFI, the AT may change the FB based on the type of heat challenge. The FB dynamics of pigs reared in constant HS differ from those of pigs reared in CHS [4]. In a CHS challenge, when the AT returns to the thermoneutrality (TN) zone, pigs increase VFI rapidly to similar levels to that of unchallenged pigs, or the VFI can temporarily exceed the normal level [5,6].

Regarding the behaviors that underlie feed intake, meal size, ingestion time per day, and occupation time of the feeding station decrease as AT increases from 19 to 29 °C, whereas the daily number of meals remains constant [7]. However, the impact on VFI is smaller for daily CHS compared to constant HS. This is because pigs show specific behavioral adaptations, such as reducing VFI during the warm periods of the day and increasing it during fresh periods, especially in the nocturnal period [5,8].

Because pigs are sensitive to their environment [9], turning the lights on and off, henceforth referred to as ‘light events’, may also alter the feeding pattern of pigs. Light events may modulate or, in some cases, block outright the circadian rhythm via a process referred to as ‘masking’ [10,11]. However, to our knowledge, no data regarding the effects after lighting events on FB of CHS pigs are available.

A better understanding of pigs’ FB during CHS and light events could provide key information for improving productivity and animal well-being, especially in tropical climate areas where pigs are usually exposed to cyclic variation in AT [12]. Accordingly, a strategy to evaluate the effects of AT and light events on FB is to compare pigs with similar body weight (BW), genetics, and sex that were raised in similar facilities, management systems, and diets but in differing AT. The aforementioned factors affect FB in different ways. Even when all these influences are kept constant, individual differences still exist [13,14], making it difficult to compare studies that do not have the same overall experimental conditions. Thus, considering that only AT differs in the current study, we hypothesized that: (I) pigs raised in CHS conditions prioritize their feed intake in the coolest hours of the day; (II) pigs overconsume during the TN period of the CHS challenge, thus having a higher meal size than their counterpart raised in TN conditions; and (III) light events modulate the meal size of pigs according to AT. Therefore, the objectives of this study were to: (i) investigate the effects of CHS compared to TN conditions on FB and (ii) evaluate the impact of light events on FB in both AT conditions of finishing pigs.

## 2. Materials and Methods

### 2.1. Data Source

The animals, facilities, and experimental procedures used in this study were previously described [1]. In this study, the feeding behavior of 90 crossbred (Landrace × Large White) finishing gilts was recorded in real-time with the aid of 10 automatic precision feeders (Automatic and Intelligent Precision Feeder—AIPF; University of Lleida, Lleida, Spain). A detailed description of the feeders is available in [15].

Pigs were allotted at the Swine Research Facilities of São Paulo State University (Unesp), Jaboticabal, SP, Brazil with 24.5 ± 2.9 kg of BW and group-housed in two similar rooms (45 pigs/room) on a full concrete floor, with a single 95 m^2^ pen each, equipped with fans and an evaporative pad cooling system (Big Dutchman, Araraquara, SP, Brazil). Pigs had free access to water via low-pressure nipple drinkers and were fed ad libitum, individually, and with diets formulated to meet or exceed their nutritional requirements according to NRC [16] recommendations. The lighting program was fixed to obtain 12 h of light as the sum of natural sunlight and LED light source (1800 Lumens). Artificial light was controlled by a timer switch from 06 to 18 h.

All pigs were fitted with an ear tag with an electronic chip (passive transponders of radio frequency identification) in the right ear, with the electronic chip number being previously registered in the feeder station system. The electronic chip provided an exclusive identification to each pig, which enabled all animals to be housed in the same pen and allowed each individual pig to be an experimental unit. Therefore, pigs had free access to any of the feeders in the room and had their individual feed intake (FI) recorded during the trial.

For feed delivery, when each pig introduced its head into the feeder, the electronic chip was identified, and when the pig triggered a push button to demand feed, the AIPF delivered a service to each request. With the aim of maintaining the services per pig per day at less than 150 requests (following the manufacturer’s recommendation), the size of the service was adjusted. The service was set to deliver the feed according to the experimental phase (EP), with 20 g during EP1 (0–20 days, 68 to 93 kg of BW) and 23 g during EP2 (21–38 days, 93 to 111 kg of BW). To avoid feed waste and ensure that pigs consumed each serving before requesting a new one, a time lag of 30 s was imposed between services. Total FI in each visit was the sum of several requests. Normally, the pigs empty the feeder after each visit, but sometimes they leave the feeder hopper with small amounts of feed (less than the service size). However, it is not significant when considering the total FI in the day.

Regardless of the facility, during the growing phase (25 to 67 kg of BW), pigs were kept in similar conditions of AT (thermoneutrality), feeding management, and diets until achieving a BW of 67.7 ± 6.2 kg when the trial started (finishing phase). Pigs remained in the experiment for 45 days, which consisted of a 7-day adaptation period to experimental diets and CHS and a subsequent 38-day experimental phase, which was divided into EP1 (Days 0 to 20) and EP2 (Days 21 to 38).

During the experimental period, in the facility with TN conditions, the AT was maintained at 22 °C, whereas in the CHS conditions, a diurnal cyclic variation in AT was set and ranged from 22 °C (20–08 h) to 35 °C (8–20 h). Thermostat-controlled heaters (Furio, São Paulo, SP, Brazil) were used to increase the AT in CHS conditions. To cool down the AT until thermoneutrality, fans, and evaporative pad cooling systems (Big Dutchman, Araraquara, SP, Brazil) were used in both conditions. An air humidifier (Hidrogiro, Ribeirão Preto, SP, Brazil) was used to increase the relative humidity of the air in the CHS condition (8–20 h) to decrease the evaporation rate and exacerbate the effect of HS. These temperatures and time periods aimed to mimic diurnal variation in ambient temperature based on studies with cyclic heat stress [12,17,18]. The AT and relative humidity were recorded daily at 30 min intervals throughout the experiment using a thermohygrometer (Instrutherm Mod. HT-70, São Paulo, Brazil).

### 2.2. Data Collection and Management

The visit of each pig to the AIPF was registered by a monitoring device that continuously and automatically recorded the start and end time of each visit (day, hour, minute, and second), the number of requests made by the pig, and the total amount of feed served in each visit. Collected information was used to build the initial database, which was initially managed by using a Microsoft Excel spreadsheet. Feeding information collected on the days that pigs were handled (i.e., blood collection, dual-energy X-ray absorptiometry scanning, as described in [1]) was removed from the dataset (3 days). In the beginning, the initial dataset had 25,704 records, which were then analyzed using R Statistical Software (version 2.14.0; R Foundation, Vienna, Austria). Initially, the dataset was evaluated by searching for the presence of outliers. Using graphical analysis, only 1 observation was removed from the dataset during the experimental period (visit longer than 3 h), which was considered a technical failure of the system. Observations of zero FI (0.06%) were excluded based on the assumption that this visit may be associated with animal–feeder interactions, in which pigs quickly introduce and remove their head from the feeder and which, hence, are recognized and recorded by the AIPF software as a visit.

### 2.3. Definition of the Meal Criterion

A visit represents every time a pig introduces its head into the feeder. However, it does not imply a meal. A meal may be defined as an accumulation of sequential visits that were separated by an interval below a calculated meal criterion. The interval represents short breaks mainly for feeder change and water intake [19], along with social interactions that can occur during breaks within a meal. Thus, expressing FB data in terms of meals is considered a more appropriate unit.

The meal criterion used in this study was based on the analysis of the starting probability. This method defines a meal based on statistical measures rather than behavioral measures, which were previously described and recommended for situations in which neither the within- nor the between-meal interval distributions are known [20]. For this purpose, the probability of the pigs starting a new feeding event within the next minute (*p*start) was calculated over the day (all dataset) and for the TN period, as well as for the HS period, and plotted against the interval between visits to the feeder. The meal criterion was estimated as the first point that minimizes the *p*start curve as previously described [20] and had been recently used in this type of dataset [8,21]. To reduce the effect of random variation in the *p*start function, a simple moving average over 21 min intervals was used.

### 2.4. Calculations and Statistical Analysis

Using the defined meal criterion (see *p*start and meal criterion section), a new database was created for group visits that consisted of the same meal, resulting in a database with 13,834 records. In the new database, intervals between meals longer than 1440 min were arbitrarily excluded and assumed to be physiologically noncompatible, but this exclusion represents 0.8% of observations from the initial database, which is a percentage that does not compromise the final analysis. Based on the data grouped into meals, the number of meals (n/pig), meal size (feed intake, g/meal), meal duration (feeder occupancy, min/meal), feed intake rate (defined as the feed intake for each minute spent feeding over a meal, g/min/meal), and the interval between meals (min) of each animal were calculated. It should be noted that pig FB changes throughout a 24 h day occur regardless of AT [8,22]. In addition, the circadian rhythm may also change FB [23]. Thus, for a more adequate approach, the day was divided into four periods: PI _(06–08 h)_, PII _(08–18 h)_, PIII _(18–20 h)_, and PIV _(20–06 h)_. PI _(06–08 h)_ and PII _(08–18 h)_ together represent the diurnal period of the day, whereas PIII _(18–20 h)_ and PIV _(20–06 h)_ are the nocturnal period. PII _(08–18 h)_ and PIII _(18–20 h)_ were defined as the HS period of the day, whereas PI _(06–08 h)_ and PIV _(20–06 h)_ were the TN period. It was assumed that PI _(06–08 h)_ and PIII _(18–20 h)_ represented the short-time effects after lighting-on and -off events, respectively.

All the data were analyzed using the MIXED procedure in SAS software (version 9.4; SAS Inst. Inc., Cary, NC, USA). The presence of outliers was evaluated through the residual analysis of data and by daily records of anomalies. The normality of studentized residuals was verified using the Cramer–von Mises test. Meal duration and interval between meals were log-transformed to adjust for lack of normality. If the data were transformed for analysis, least squares means were back-transformed to the original scale for reporting purposes. Each pig was considered an experimental unit. The model included the fixed effects of temperature (T), period of the day (P), experimental phase (EP), and their interactions (T × P, T × EP, P × EP, and EP × T × P) as follows:

Yijmk = µ + Ti + Pj + EPm + (T × P)ij + (T × EP)im + (P × EP)jm + (T × P × EP)ijm + eijmk(1)

where Yijmk is the observed variable (observation for the kth pig from the (i,j,m)th cell), μ is the overall mean, Ti is the effect of the ith level of the T factor, Pj is the effect of the jth level of the P factor, EPm is the effect of the mth level of the EP factor, and their interactions (that is, ith level of T with the jth level of P, (T × P)ij; ith level of T with the mth level of EP, (T × EP)im; jth level of P with the mth level of the EP, (P × EP)jm; and ith level of T with the jth level of P with the mth level of the EP, (T × P × EP)ijm). The eijmk was considered the random error of the kth observation from (i,j,m)th.

The repeated measurement option was used with a compound symmetry covariance structure to account for the animal effect over sampling time, and the initial body weight was included as a covariate. Preplanned contrast comparisons were performed to evaluate the effects of light-on (C_1_, PI _(06–08 h)_ × [PIII _(18–20 h)_ + PIV _(20–06 h)_]) and light-off events (C_2_, PIII _(18–20 h)_ × [PI _(06–08 h) +_ PII _(08–18 h)_]). A third contrast (C_3_, [PI _(06–08 h)_ + PII _(08–18 h)_] × [PIII _(18–20 h)_ + PIV _(20–06 h)_]) was performed to compare the diurnal and nocturnal periods. Differences were considered significant if *p* ≤ 0.05. When there was a P × EP × P interaction effect (*p* < 0.05), adjusted means of P were compared in each T within each EP using the Tukey–Kramer test. When there was a T × P interaction effect (*p* < 0.05), adjusted means of P were compared in each T using the Tukey–Kramer test. Likewise, when there was a P × EP interaction effect (*p* < 0.05), adjusted means of P were compared in each EP. Finally, a *t*-test was used to compare the AT and air relative humidity differences between rooms.

## 3. Results

### 3.1. General Observations

**Figure 1:**
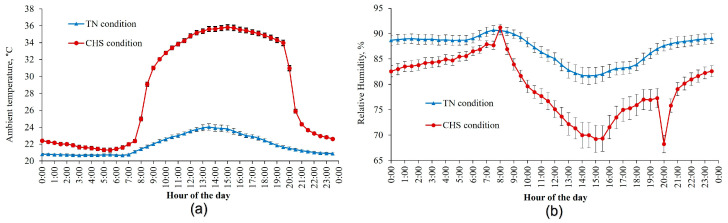
Average ambient temperature (**a**) and air relative humidity (**b**) were measured in thermoneutral (TN) and cyclic heat stress (CHS) conditions, with the aid of a thermohygrometer, at 30 min intervals, during the experimental days. Each vertical bar represents the standard error of the mean.

The average AT and air relative humidity experienced by the animals inside each room (TN and CHS) during the experiment are shown in Figure 1. The AT differed between rooms (*p* < 0.01) on a 24 h average basis. Overall, the AT in TN conditions averaged 21.9 ± 0.4 °C, whereas in CHS, the AT ranged from 22.3 ± 0.2 °C (TN period) to 33.8 ± 0.3 °C (HS period). The relative humidity also differed between rooms (*p* < 0.01) and averaged 87 ± 1.7% and 79.6 ± 2.7%, for TN and CHS rooms, respectively.

Detailed information on growth performance and carcass composition was reported in our previous study [1], and individual pig behavior profiles showing the heterogeneity in FB response are available in Supplementary Figure S1.

### 3.2. pstart and Meal Criterion

**Figure 2:**
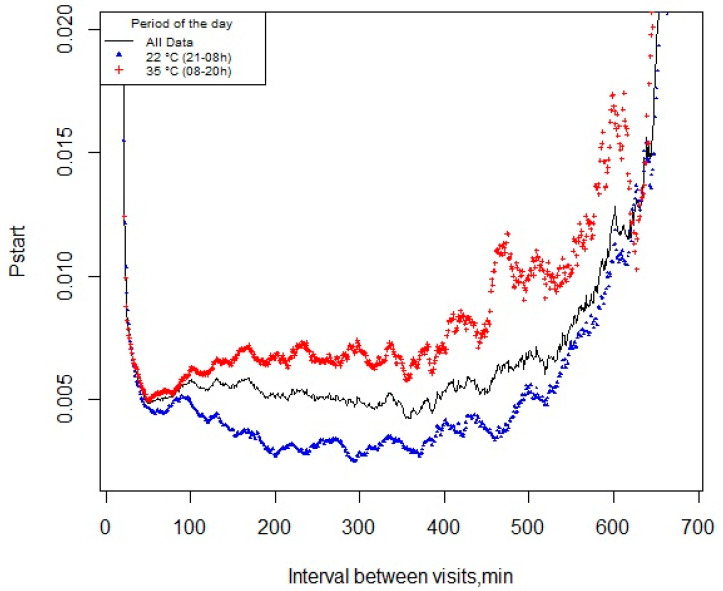
Probability of pigs starting a new feeding event (*p*start) within the next minute since the last visit over the course of the day, or during exposure to cyclic high ambient temperature (35 °C, 0800–2000 h) or thermoneutrality (22 °C, 2000–0800 h).

A total of 24,128 visit records were used to calculate the *p*start function. There was no difference in determining the meal criterion using the *p*start methodology for exposure to different temperatures. Thus, the probability of pigs starting a new feeding event within the next minute after the last visit—over the course of the day, during exposure to CHS (35 °C) or TN (22 °C)—was 49 min (Figure 2).

### 3.3. Feeding Behavior Responses

Overall, the feed intake (meal size × number of meals) of pigs throughout the experiment on a 24 h basis was 2416 g and 2268 g for the TN and CHS conditions, respectively, indicating that CHS reduced the FI by 6.13%.

**Figure 3:**
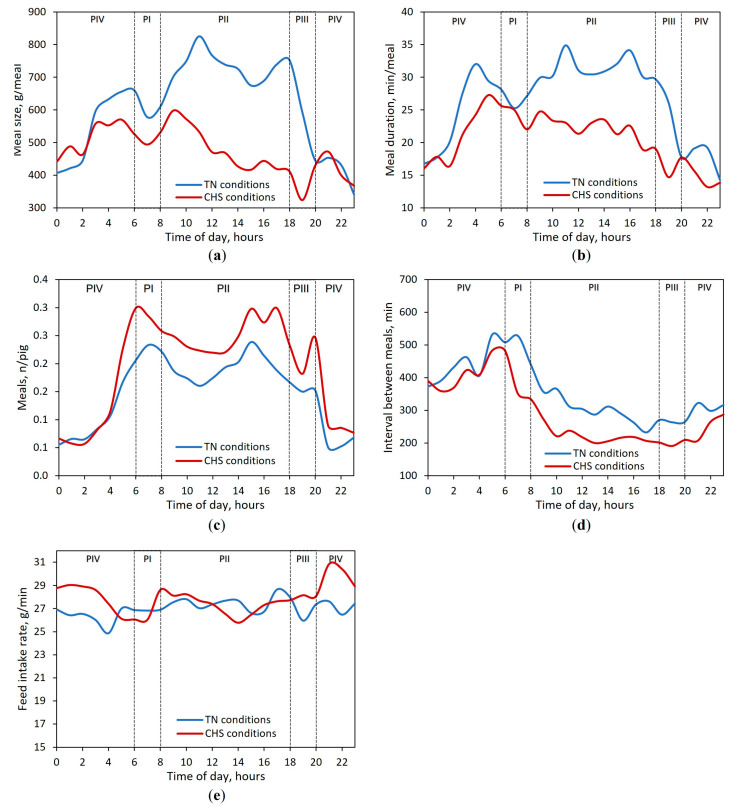
Circadian variation in (**a**) meal size, (**b**) meal duration, (**c**) number of meals, (**d**) interval between meals, and (**e**) feed intake rate for finishing pigs reared in thermoneutrality (TN, 24 h at 22 °C) or cyclic heat stress (CHS, 12 h at 22 °C, 20–08 h, and 12 h at 35 °C, 08–20 h) conditions for 38 days of the experimental period. Periods of the day: PI _(06–08 h)_; PII _(08–18 h)_; PIII _(18–20 h)_; PIV _(20–06 h)_. Average data of 45 pigs per AT condition throughout 24 h day, regardless of the experimental phase. The lighting program was fixed to 12 h of artificial light (06 to 18 h).

The meal size continuously recorded in TN and CHS conditions followed a circadian pattern with typical diurnal FB (Figure 3a). Interestingly, the meal size of the pigs decreased during the first hour after the light had been switched on, independent of the AT. In the TN condition, the hourly meal size peaked twice over the diurnal period, whereas in the CHS condition, it peaked once. Under TN conditions, the first peak took place 5 h after the light had been switched on (11 h), whereas the second peak took place before the end of the diurnal period (18 h). In the CHS condition, the diurnal peak took place 3 h after the light had been switched on (9 h). When the lights were switched off, pigs in the TN condition started to decrease the meal size until 23 h, whereas in the CHS condition, an increase in meal size was observed between 19 and 21 h. The meal duration had a similar trend to that of meal size (Figure 3b).

Most feed intake, 77.2% and 73.2%, and meals, 71.6% and 72.2%, were observed between 6 and 18 h in the TN and CHS conditions, respectively, which corresponded to the light-on period (Figure 3c). Irrespective of AT, the interval between meals started to decrease during the diurnal period, right after the light had been switched on (Figure 3d). Overall, the feed intake rate was quite similar throughout the 24 h day (Figure 3e).

Feeding behavior responses of the pigs are presented in Table 1. Residuals of feed intake rate, meal size, and number of meals were normally distributed, whereas the residuals of meal duration and the interval between meals were log-transformed to adjust for lack of normality. Initial BW as a covariate was significant for all studied variables (*p* < 0.01). Overall, responses were affected by P and EP, except the number of meals (*p* < 0.01).

Interactions between P × T were detected for feed intake rate (Table 1). In the CHS condition, pigs had the highest feed intake rate during PIII _(HS, 18–20 h)_, the lowest feed intake rate during the diurnal period, from 06 to 18 h (PI _(TN, 06–08 h)_ + PII _(HS, 08–18 h)_), and an intermediate value during PIV _(TN, 20–06 h)_ (*p* < 0.01, P × T). However, there was no difference in the feed intake rate in the TN condition during the periods of the day.

A P × EP interaction was detected for feed intake rate (Table 2). During EP1, pigs had a greater feed intake rate during PIII _(18–20 h)_ than during the diurnal periods, whereas no effect of P was observed in EP2 (*p* = 0.01, P × EP). In contrast, as expected, regardless of the period of the day, pigs during EP2 had a greater feed intake rate than those during EP1 (*p* = 0.01, data not shown).

The interaction between EP × T × P was significant for meal size, meal duration, and the interval between meals (Table 3). In terms of period effects within each temperature, in EP1 (Days 0 to 20), in TN conditions, pigs had the highest meal size and duration in PII _(08–18 h)_ whereas in the other period, similar values were observed (*p* < 0.01). Under CHS conditions, pigs had a decrease in meal size in PIII _(18–20 h),_ whereas in the remaining period, similar values were observed (*p* < 0.01). The highest meal duration was observed in PII _(08–18 h),_ and the lowest meal duration was observed in PIII _(18–20 h)_ (*p* < 0.01). It should be noted that for both ATs, pigs had a decrease (*p* < 0.05) in meal size and duration from PII _(08–18 h)_ to PIII _(18–20 h)_ (after turning off the light). However, only for the CHS condition did pigs show an increased (*p* < 0.05) meal size and duration from PIII _(HS, 18–20 h)_ to PIV _(TN, 20–06 h)_. In both conditions, pigs had a lower interval between meals from 08 to 20 h (PII _(08–18 h)_ + PIII _(18–20 h)_) (*p* < 0.01). In EP2 (Days 21 to 38), pigs in TN conditions had the highest and lowest meal size and duration in PII _(08–18 h)_ and PIII _(18–20 h)_, respectively (*p* < 0.01). The highest interval between meals was observed in PIV _(20–06 h)_, the lowest in PI _(06–08 h)_, and intermediate values from 08 to 20 h (PII _(08–18 h)_ + PIII _(18–20 h)_) (*p* < 0.01). Under CHS conditions, the lowest meal size and duration were observed in PIII _(HS, 18–20 h)_. Meal duration in the remaining periods was similar (*p* > 0.05). For both AT, pigs had a decrease (*p* < 0.05) in meal size and duration from PII _(08–18 h)_ to PIII _(18–20 h)_ (after turning off the light) and an increase (*p* < 0.05) in meal size and duration from PIII _(18–20 h)_ to PIV _(20–06 h)_. The highest interval between meals was observed in PIV _(20–06 h)_, whereas the lowest was observed in PII _(HS, 08–18 h)_ (*p* < 0.01).

Regarding temperature conditions (TN × CHS) within each period, several changes were observed. Overall, the CHS temperature reduced the values for all analyzed variables in all periods (*p* < 0.01), except for the interval between meals in PIV _(20–06 h)_ during EP1 and in PIII _(18–20 h)_ during EP2 (*p* > 0.05).

Switching on the lights stimulated the feed intake of CHS pigs by 12.9% (*p* < 0.01) in comparison to the entire light-off period (C1, Table 4), whereas no effect was observed for TN pigs (4.2%, *p* > 0.05). Alternatively, switching off the lights induced a reduction in the feed intake of pigs, and hence in meal size by 17.8% for TN and 19.1% for CHS conditions related to the entire light-on period (C2, *p* < 0.01). During the entire light-on period, the meal size was 16.8% and 9.3% greater in TN and CHS, respectively, compared to the light-off period (C3, *p* < 0.01).

## 4. Discussion

The current literature evaluating the effects of CHS and pig circadian rhythm on FB traits is scarce. Understanding the impact of AT variation and light events may aid in explaining the physiology and growth response of pigs under CHS conditions. Therefore, we hypothesized that pigs reared in CHS conditions prioritize feed intake in the coolest hours of the day, whereas light events alter the FB of pigs under cyclic AT variation over the day. Our major and original finding was that even when adapted, pigs in CHS conditions had a different FB when compared to TN pigs. Therefore, this FB response may help to explain differences in growth performance and body composition for CHS pigs, as previously reported [1].

Feeding behavior may be affected by many factors such as sex [24], breed [8], body weight [25], circadian rhythm [23], ambient temperature [7], type of heat challenge (warm constant × cyclic temperatures; [4]), origin, age [26], diet, and handling. Therefore, a single experiment with pigs which have similar characteristics and differ only in the AT condition, as described in the present study, seems to be more suitable for evaluating the impact of AT on FB. In the current study, it was possible to measure the feed intake over different periods of the day. Period II _(08–18 h)_ and PIV _(20–06 h)_ represented more than 80% of the diurnal and nocturnal period of a 24 h day. Thus, we used both periods to evaluate the dynamic impact of HS on feed intake. Alternatively, PI _(06–08 h)_ and PIII _(18–20 h)_ represented the period after switching the lights on and off, respectively, and herein, these periods were used to compare changes that occurred in the FB due to light events.

Feed intake rate increases over time because body size and oral capacity become larger, which allows pigs to take larger bites and eat more feed in a shorter time [6]. This supports the feed intake rate observed in the current study.

Although changes in feed intake rate were observed between periods of the day in EP1, no differences were detected during EP2. We believe that changes in feed intake rate in EP1 may be more closely associated with HS impact once a reduced feed intake is observed in pigs during the warmer period of the day. Because pigs under CHS conditions were in an adaptation process during EP1, the higher feed intake rate for CHS pigs in some specific periods of the day may have contributed to the overall mean, which could have triggered the interaction effects. This concept is supported by the higher feed intake rate observed during the nocturnal period (the coolest part of the day). Alternatively, as long as pigs became more adapted to the CHS condition, they normalized their feed intake rate over the day, which resulted in a lack of period effect in the EP2. Our outcomes agree with those of Fraga et al. [8], who also did not observe changes in feed intake rate throughout the day for pigs (22 kg to 105 kg of body weight) raised under a similar CHS protocol. To maintain a constant feed intake rate throughout the day under CHS conditions, pigs increase the meal size, which implies an increase in meal duration [27].

Moreover, an interaction between P × T was observed for feed intake rate for pigs in different AT conditions. In our study, as expected for pigs reared under unrestricted conditions [27], feed intake rate was kept relatively constant throughout the day for TN pigs. However, it should be noted that for pigs under CHS conditions, feed intake rate increased when the lights were switched off, whereas for TN, no changes were observed. Such behavior highlights the fact that the light events modulated pigs’ FB according to the AT. As widely described in the literature [28], pigs in HS conditions reduce physical activity, which probably disrupted the normal feed intake rate of pigs in the current study. The increase in feed intake rate associated with the light-off event seems to be an attempt of pigs to overcome the lower feed intake rate during the HS diurnal period. Considering that a light-off event would be expected to lower the feed intake (positively correlated with feed intake rate, [29]), the opposite behavior observed herein might be understood as a feeding motivation for pigs to try to compensate for the HS diurnal impact. In other words, the light-off event may have triggered some competition for feeder access, because group-housed pigs in restricted conditions increase feed intake rate as a mechanism to maintain meal size levels [6].

The current study showed that meal size, duration, and interval between meals were affected by AT and P, which varied according to the EP. These variables are expected to be correlated because when the meal size increases, the meal duration increases simultaneously because the feed intake rate is relatively constant throughout the day for pigs under unrestricted conditions [27]. Irrespective of AT, as long as pigs increase the meal size and duration while keeping a constant number of daily meals, the interval between meals decreases [7]. According to the concept of satiety, when a pig is satiated at the end of a meal, the probability of starting a new meal soon is low but increases over time [21]. This finding supports the reduction in meal size and duration and the increased interval between meals observed for CHS pigs during the nocturnal compared to the diurnal period, irrespective of EP.

Regarding the P effect within each EP, the pattern of meal size, meal duration, and the interval between meals for CHS pigs changed in comparison to TN pigs. During EP1, pigs in CHS conditions had similar meal sizes in the diurnal and nocturnal periods. Interestingly, during EP2, the meal size during the nocturnal period exceeded that observed for the diurnal period, whereas no changes in meal duration were observed. Because the animals had a constant number of daily meals, pigs increased the meal size in the same meal duration, which implied a higher feed intake. Taken together, these points reinforce the concept that pigs raised under CHS use mechanisms associated with changes in the FB to adapt to AT conditions and to maintain a constant number of meals throughout the day.

Irrespective of the EP, our study demonstrated that pigs raised in TN reduced their meal size during the nocturnal period. In contrast, pigs raised in CHS had a similar meal size during the diurnal and nocturnal periods in EP1, whereas it was greater during the nocturnal period in EP2. Thus, our data support the hypothesis that the pigs tried to compensate for the impact of CHS by eating more during the TN period of challenge. These results agree with previous studies that also evaluated pigs exposed to CHS [5,8]. However, it should be noted that the compensatory response was not enough to allow a similar meal size compared to the pigs under TN conditions. Although pigs exhibit adaptive FB, the time required for feed intake recovery after exposure to HS to reach a similar level to that of TN pigs can vary between 2 and 5 days [30,31,32]. However, factors related to the physical capacity of the pigs´ digestive tract and the impact of the consecutive daily heat challenge might not have allowed a full recovery of feed consumption in the current study. Thus, changes in the FB of pigs under CHS observed in the current study may be associated with the adaptation process to the cyclical AT condition over the day. This adaptation response may explain the differences in growth performance, as well as body composition, which was previously reported [1].

Pigs predominantly exhibit a diurnal feeding consumption, with most meals (73% on average) consumed between 06 and 18 h [23,25]. Similar values were observed in our experiment during the diurnal period (71.6% for TN and 72.2% for CHS). In the TN condition, the hourly meal size peaked twice over the diurnal period (at approximately 11 and 18 h), suggesting that this response may be related to the lighting program used [6]. In contrast, the meal size of pigs under CHS conditions, in addition to being lower, ended up in just a single peak at the beginning of the HS period (at approximately 9 h). This response may be an adaptation mechanism of pigs to HS in an attempt to decrease heat production and, consequently, the amount of heat that needs to be dissipated into the environment [33,34].

According to our results, an interesting finding is that the meal size of CHS pigs decreased during the first hour after the light-off event and started to increase shortly thereafter. In contrast, when the light was switched off, a huge drop in meal size was observed for pigs under TN conditions, and this response was maintained until the end of the day. This behavior confirms the strong influence of lighting events on circadian rhythm, which drive an increased activity at a period of the day and a decrease in activity at another [11] for TN pigs. In contrast, for CHS pigs, after the first hour of darkness, the meal size continuously increased for 2 h. This indicates that although the nocturnal period had begun, the pigs continued to ingest feed, which suggests that CHS conditions affected the pigs´ circadian rhythm. This response coincides also with a higher observed feed intake rate.

Our data showed that the light events change the meal size of pigs. However, the changes were different in each AT condition. Pigs in TN conditions increased the meal size by 4.2% and reduced it by 17.8% during the light-on and -off events, whereas pigs in CHS conditions increased the meal size by 12.9% and reduced it by 19.1% during the light-on and -off events, respectively. The higher improvement (in %) in meal size for CHS than for TN pigs during a light-on event may be associated with the reduced feed intake during the light-off period. The calorie restriction might increase motor activity [35]. In addition, a change in FB is also part of the metabolic cues associated with calorie restriction, which is reported to affect the suprachiasmatic nuclei of the hypothalamus and, hence, the synchronization to light [35]. Thus, the FB of CHS was modulated by light events when compared to TN pigs.

## 5. Conclusions

In conclusion, the outcomes showed that pigs raised under CHS changed their FB to maintain a constant number of meals throughout the day. However, despite our initial hypothesis that pigs could prioritize their feed intake in the coolest hours of the day, our results demonstrate that pigs did not fully compensate for feed intake depression caused by the HS. Despite both AT being affected by light events, CHS pigs had a greater increase in feeding activity than TN pigs when the light was switched on. The understanding of the pig responses to temperature variation is of the utmost importance in establishing strategies to improve pig production under warm climates. Because our results showed that light events affect feeding behavior under daily cyclic high-ambient-temperature conditions, research on diet modulation over the day is encouraged.

## Figures and Tables

**Table 1:** Feeding behavior of finishing pigs exposed to thermoneutrality or cyclic heat stress conditions, according to the period of the day ^1,2^.

	Temperature (T)											
	Thermoneutrality				Cyclic Heat Stress				ANOVA *p*-Value ^4^			
**Period of the Day (P)**	**PI _(06–08 h)_**	**PII _(08–18 h)_**	**PIII _(18–20 h)_**	**PIV _(20–06 h)_**	**PI _(TN, 06–08 h)_**	**PII _(HS, 08–18 h)_**	**PIII _(HS, 18–20 h)_**	**PIV _(TN, 20–06 h)_**	**P × T**	**P × EP**	**T × EP**	**P × EP × T**
Feed intake rate, g/min ^5^	27.4 (0.40)	27.9 (0.32)	27.4 (0.45)	27.4 (0.35)	27.6 ^b^ (0.39)	27.5 ^b^ (0.32)	28.6 ^a^ (0.41)	27.9 ^ab^ (0.34)	<0.01	0.01	0.89	0.30
Meal size ^6^, g/meal	605 (15)	751 (12)	557 (18)	604 (14)	516 (15)	483 (12)	404 (16)	510 (13)	<0.01	<0.01	0.83	<0.01
Meal duration ^3,6^, min/meal	25.9 (0.87)	31.5 (0.59)	22.4 (1.03)	26.0 (0.72)	23.3 (0.83)	22.0 (0.56)	16.4 (0.91)	21.5 (0.66)	<0.01	0.02	0.50	<0.01
Interval between meals ^3,6^, min	507 (14)	310 (11)	300 (15)	504 (12)	350 (13)	224 (11)	219 (14)	421 (12)	<0.01	<0.01	0.73	0.01
Number of meals, n/pig	0.23 (0.01)	0.19 (0.01)	0.15 (0.01)	0.09 (0.01)	0.27 (0.01)	0.25 (0.01)	0.21 (0.01)	0.12 (0.01)	0.17	0.71	0.82	0.81

¹ Data after application of meal criterion defined as 49 min and expressed per hour of the day since each periods of the day had a different duration. The thermoneutrality condition was 24 h at 22 °C, whereas the cyclic heat stress condition was set at 12 h at 22 °C, 20–08 h, and 12 h at 35 °C, 8–20 h. ² Least squares means and standard error (SE). ³ Meal duration and the interval between meals were log-transformed for statistical analysis and back-transformed to the original scale for reporting purposes. ^4^ Probability of periods of the day (n = 4; P), temperature (n = 2; T), experimental phase (n = 2; EP), and interactions between P × T, P × EP, T × EP, and P × EP × T. For variables whose interaction was significant, average values were analyzed by Tukey’s test. Initial BW as a covariate was significant for all variables; *p* < 0.01. ^5^ The P × EP interaction for feed intake rate is presented in Table 2. ^6^ The P × EP × T interaction for feed intake, meal duration, and the interval between meals are presented in Table 3. ^a,b^ Values within a row and temperature, with different superscripts, differed during the period of the day (P × T, *p* < 0.01).

**Table 2:** Feed intake rate of finishing pigs according to the period of the day in each experimental phase ^1,2^.

	Period of the Day (P)				ANOVA
Experimental Phase (EP)	PI _(06–08 h)_	PII _(08–18 h)_	PIII _(18–20 h)_	PIV _(20–06 h)_	*p*-Value ^3^
Experimental phase ^1^ (0–20 days)					
Feed intake rate, g/min	25.6 ^b^ (0.40)	25.7 ^b^ (0.32)	26.9 ^a^ (0.42)	26.2 ^ab^ (0.34)	<0.01
Experimental phase ^2^ (21–38 days)					
Feed intake rate, g/min	29.3 (0.40)	29.7 (0.32)	29.3 (0.44)	29.2 (0.35)	0.22

^1^ Data after application of meal criterion defined as 49 min and expressed per hour of the day since each periods of the day had a different duration. ^2^ Least squares means and standard error (SE), represent the average feed intake rate obtained from pigs raised under thermoneutrality (24 h at 22 °C) and cyclic heat stress conditions (12 h at 22 °C, 20–08 h, and 12 h at 35 °C, 8–20 h). ^3^ Statistical analyses between periods of the day in each EP. ^a,b^ Values within a row with different superscripts differed between periods of the day in each EP (*p* < 0.05) according to Tukey’s test.

**Table 3:** Feeding behavior of finishing pigs exposed to thermoneutrality conditions or cyclic heat stress conditions, according to the period of the day in each experimental phase ^1,2^.

	Temperature (T)								
	Thermoneutrality				Cyclic Heat Stress				ANOVA
Period of the Day (P)	PI _(06–08 h)_	PII _(08–18 h)_	PIII _(18–20 h)_	PIV _(20–06 h)_	PI _(TN, 06–08 h)_	PII _(HS, 08–18 h)_	PIII _(HS, 18–20 h)_	PIV _(TN, 20–06 h)_	*p*-Value ^4^
Experimental phase ^1^ (0–20 days)									
Meal size, g/meal	587 ^b^ (22)	686 ^a^ (17)	581 ^b^ (24)	569 ^b^ (19)	484 ^a^ (21)	470 ^a^ (17)	387 ^b^ (22)	460 ^a^ (18)	<0.01
Meal duration ^3^, min/meal	27.0 ^b^ (1.22)	31.6 ^a^ (0.82)	24.1 ^b^ (1.43)	24.7 ^b^ (0.96)	23.7 ^ab^ (1.15)	23.1 ^a^ (0.79)	16.4 ^c^ (1.24)	20.7 ^b^ (0.93)	<0.01
Interval between meals ^3^, min	507 ^a^ (19)	289 ^b^ (15)	303 ^b^ (21)	432 ^a^ (17)	381 ^b^ (18)	222 ^c^ (15)	204 ^c^ (19)	393 ^a^ (16)	<0.01
Experimental phase ^2^ (21–38 days)									
Meal size, g/meal	624 ^b^ (22)	816 ^a^ (17)	534 ^c^ (25)	640 ^b^ (20)	547 ^a^ (21)	496 ^b^ (17)	420 ^c^ (23)	559 ^a^ (18)	<0.01
Meal duration, min/meal	24.7 ^b^ (1.25)	31.5 ^a^ (0.84)	20.7 ^c^ (1.50)	27.2 ^b^ (1.07)	23.0 ^a^ (1.19)	20.8 ^a^ (0.80)	16.5 ^b^ (1.33)	22.3 ^a^ (0.95)	<0.01
Interval between meals, min	507 ^b^ (19)	331 ^c^ (15)	297 ^c^ (22)	576 ^a^ (18)	319 ^b^ (19)	226 ^c^ (15)	234 ^bc^ (20)	448 ^a^ (16)	<0.01

^1^ Data after application of meal criterion defined as 49 min and expressed per hour of the day since each periods of the day had a different duration. The thermoneutrality condition was 24 h at 22 °C, whereas the cyclic heat stress condition was set at 12 h at 22 °C, 20–08 h, and 12 h at 35 °C, 8–20 h. ^2^ Least squares means and standard error (SE). ^3^ Meal duration and the interval between meals were log-transformed for statistical analysis and back-transformed to the original scale for reporting purposes. ^4^ Statistical analyses between periods of the day in each experimental phase for control and challenged barn. ^a–c^ Values within a row with different superscripts differed between periods of the day in each barn (*p* < 0.05) according to Tukey’s test.

**Table 4:** Meal size of finishing pigs exposed to thermoneutrality conditions or cyclic heat stress conditions, according to the period of the day ^1,2^.

	Period of the Day (P)				ANOVA *p*-Value ^3^		
Temperature (T)	PI _(06–08 h)_	PII _(08–18 h)_	PIII _(18–20 h)_	PIV _(20–06 h)_	C_1_	C_2_	C_3_
Thermoneutrality							
Meal size, g/meal	605 (15)	751 (12)	557 (18)	604 (14)	0.15	<0.01	<0.01
Cyclic heat stress							
Meal size, g/meal	516 (15)	483 (12)	404 (16)	510 (13)	<0.01	<0.01	<0.01

^1^ Data after application of meal criterion defined as 49 min and expressed per hour of the day since each periods of the day had a different duration. ^2^ Least squares means and standard error (SE) represent the average meal size obtained from pigs raised under thermoneutrality (24 h at 22 °C) and cyclic heat stress conditions (12 h at 22 °C, 20–08 h, and 12 h at 35 °C, 8–20 h). ^3^ C_1_, PI _(06–08 h)_ × [PIII _(18–20 h)_ + PIV _(20–06 h)_]; C_2_, PIII _(18–20 h)_ × [PI _(06–08 h) +_ PII _(08–18 h)_]; C_3_, [PI _(06–08 h)_ + PII _(08–18 h)_] × [PIII _(18–20 h)_ + PIV _(20–06 h)_].

## Data Availability

The data supporting the conclusions of this article will be made available by the authors upon request.

## Supplementary Materials

The following supporting information can be downloaded at: https://www.mdpi.com/article/10.3390/ani13050908/s1, Figure S1: Individual pig behavior profiles of pigs during the experimental period throughout 24 h day according to ambient temperature (Thermoneutral vs. Cyclic heat stress) and periods of the day: PI _(06–08 h)_; PII _(08–18 h)_; PIII _(18–20 h)_; PIV _(20–06 h)_.
