# Supplementary material for: Feeding Behavior of Finishing Pigs under Diurnal Cyclic Heat Stress
Source: Animals (Basel). 2023 Mar 2;13(5):908. doi: 10.3390/ani13050908 (PMC10000165; doi:10.3390/ani13050908)
Supplement: Supplementary file 1 [file animals-13-00908-s001.zip › animals-2204102-supplementary.pdf]

Feeding Behavior of Finishing Pigs under Diurnal Cyclic Heat Stress

Marllon José Karpeggiane de Oliveira [1], Marcio Valk [2], Antônio Diego Brandão Melo [1], Danilo Alves Marçal [1], Cleslei Alisson Silva [1], Graziela Alves da Cunha Valini [1], Pedro Righetti Arnaut [1], Joseane Penteado Rosa Gonçalves [1], Ines Andretta [3] and Luciano Hauschild [1,*]

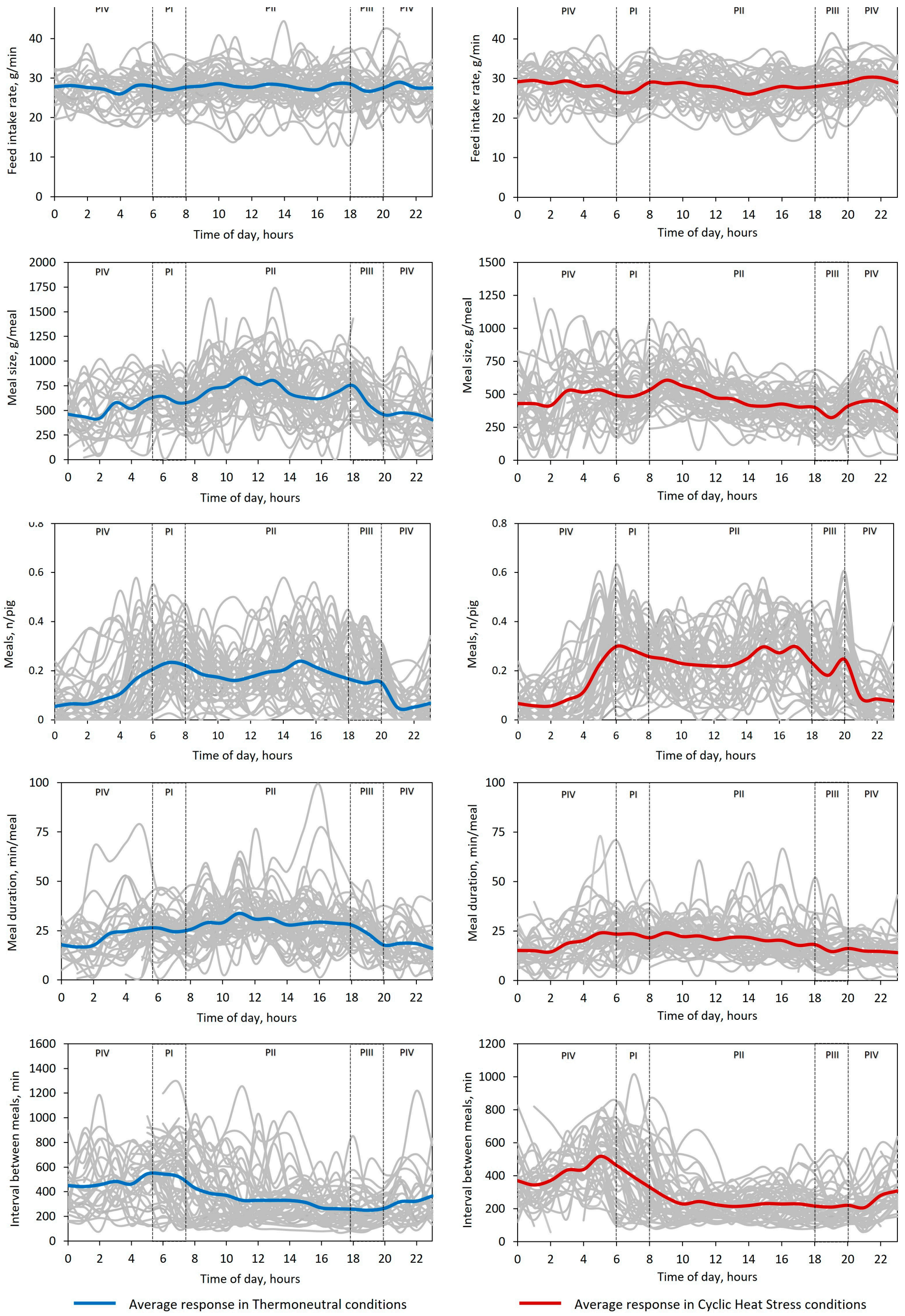

Supplementary Figure S1. Individual pig behavior profiles of pigs during the experimental period throughout 24 h-day according to ambient temperature (Thermoneutral vs. Cyclic heat stress) and periods of the day: PI (06-08 h); PII (08-18 h); PIII (18-20 h); PIV (20-06 h). The thermoneutrality condition was 24 h at 22°C, while the cyclic heat stress condition was 12 h at 22 °C, 2000-0800 h, and 12 h at 35 °C, 0800-2000 h.
